# Case report: Long-term follow-up of two patients with LHON caused by DNAJC30:c.152G>A pathogenic variant-case series

**DOI:** 10.3389/fneur.2022.1003046

**Published:** 2022-10-28

**Authors:** Sanja Petrovic Pajic, Martina Jarc-Vidmar, Ana Fakin, Maja Sustar Habjan, Jelka Brecelj, Marija Volk, Ales Maver, Borut Peterlin, Marko Hawlina

**Affiliations:** ^1^Eye Hospital, University Medical Centre Ljubljana, Ljubljana, Slovenia; ^2^Clinic for Eye Diseases, University Clinical Centre of Serbia, Belgrade, Serbia; ^3^Clinical Institute of Genomic Medicine, University Medical Centre Ljubljana, Ljubljana, Slovenia

**Keywords:** arLHON, DNAJC30:c.152G>A gene, 152 A>G (p.Tyr51Cys) pathogenic variant, visual acuity improvement, perimetry improvement, color vision improvement, recessive optic neuropathy

## Abstract

**Background:**

We present the disease course and long-term follow-up of two patients who were phenotypically diagnosed with atypical Leber Hereditary Optic Neuropathy (LHON) 14 and 12 years ago, respectively, whereby whole exome sequencing revealed recently described recessive DNAJC30:c.152G>A 152 A>G (p.Tyr51Cys) homozygous pathogenic variant with significant spontaneous visual acuity recovery in one.

**Case presentation:**

Two presented unrelated males with atypical LHON with sequential visual acuity (VA) loss were followed for many years. Both patients had negative family history. At the presentation at ages 17 (Case 1) and 18 years (Case 2), both had reduced visual acuity (Snellen): (Case 1) right eye (RE):CF 3m, left eye (LE):0.6, (Case 2) RE:0.2, LE:0.15; and color vision (Ishihara): (Case 1) 1/15 and 13/15; (Case 2) 2/15 and 3/15. Both had hyperemic optic disks (PNO) and central scotoma in their visual fields. Electrophysiology in the acute phase showed reduced and delayed visually evoked potentials (VEP) P100 in both patients, with reduced N95 amplitude in Case 2, and initially normal N95 amplitude in Case 1. Fluorescein angiography showed no early leakage with some late pooling at optic disks. Extensive clinical workout, including brain magnetic resonance imaging (MRI), aquaporin 4 (Aq4), and anti-myelin oligodendrocyte protein (anti-MOG) antibodies, was negative. Intravenous corticosteroids did not improve vision. Both experienced further deterioration several months after the onset accompanied by thinning of the peripapillary retinal nerve fiber layer (RNFL). Genetic testing for typical LHON pathogenic variants and whole mitochondrial DNA (mtDNA) sequencing was negative. 1 year after the onset, modest VA improvement began in Case 2 and continued over the next 3 years. VA improved bilaterally to 0.7, color vision 15/15, and islands of vision appeared within the visual field scotoma. VEP P100 peak time shortened, and amplitude increased, despite further RNFL thinning on optical coherent tomography (OCT). The patient's visual function remained stable during the entire 12-year follow-up period. Case 1 experienced modest VA improvement to 0.1 with some improvement in the visual field seven years after the disease onset, remaining stable during the entire 14-year follow-up period. VEP P100 wave remained undetectable.

**Conclusions:**

Presented are two autosomal recessive LHON (arLHON, OMIM:619382) cases with the same DNAJC30:c.152G>A pathogenic variant and different degrees of spontaneous visual recovery despite progressive RNFL thinning during a long-term follow-up. This mutation should be screened in every atypical LHON patient.

## Introduction

Autosomal recessive homozygous pathogenic variants in a J domain of the DNAJC30:c.152G>A gene have recently been confirmed as causative for the development of the LHON-like phenotype. Due to a novel distinct inheritance way, this phenotype is named autosomal recessive LHON (arLHON) (1). Phenotypically there is no significant difference to mitochondrial LHON (mtLHON) with the pathognomonic triad of ophthalmological features (circumpapillary telangiectatic microangiopathy, optic disc hyperemia without leakage on fluorescein angiography, and the subacute phase swelling (pseudoedema) of the retinal nerve fiber layer (RNFL)) as leading features (2). The male prevalence is even higher (10:1) than in mtLHON. The main difference is the earlier age of the disease onset and the higher visual acuity recovery rate reported in arLHON (1).

## Case presentation

Long-term follow-up of two patients harboring homozygous DNAJC30:c.152G>A:c.152A>G (p.Tyr51Cys) pathogenic variant is presented. The affected patients had a detailed diagnostic workup to exclude compressive, demyelinating, inflammatory, infective, infiltrative, or toxic causes. MRI of the brain was normal. Ophthalmological examinations were performed at the presentation as well as during follow-up periods and included: best corrected visual acuity (Snellen), color vision (Ishihara plates), visual field examination (Goldmann or Octopus perimetry), fluorescein angiography (FA), and microperimetry and electrophysiology testing. A detailed methodology is described in Supplementary Table 1. Patients provided written informed consent according to regulations of the University Medical Centre Ljubljana; the use of clinical data was approved by the National Committee for Medical Ethics (No:0120-626/2019/5). All patients received all examinations as a routine diagnostic workup.

### Case 1

**Figure 1:**
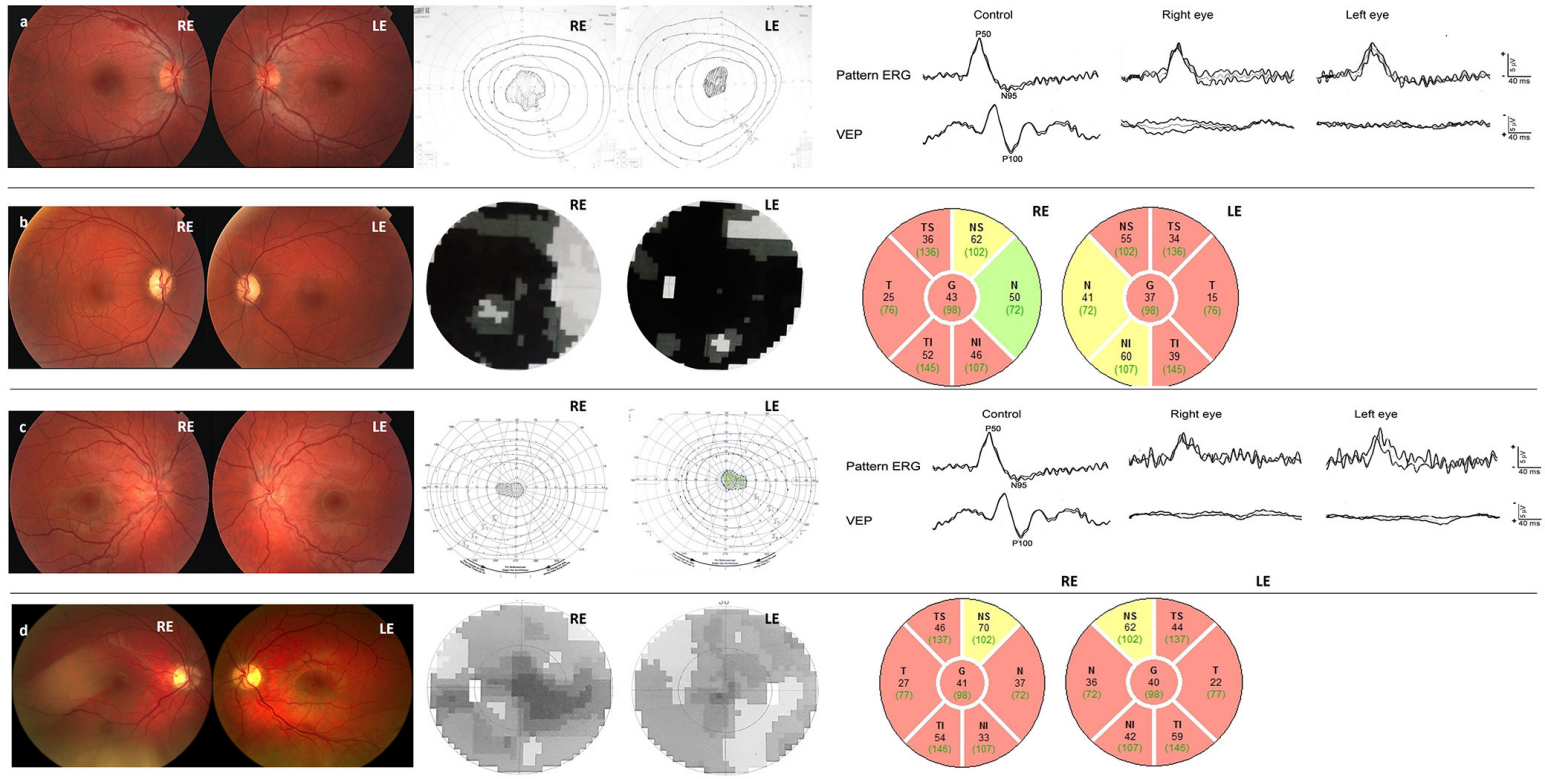
**(a)** Case 1 Fundi at the disease onset: hyperemic optic disks, without vessel tortuosity, and with intraretinal hemorrhage at the superior arcade, central scotoma in the visual field, and normal PERG, delayed and decreased VEP 100. **(b)** Case 1 at the last check-up 14 years after the disease onset: pale and atrophic optic disks, small fenestrations in the central scotoma in the visual field, and thinning of the peripapillary RNFL. **(c)** Case 2 Fundi at the disease onset: Hyperemic optic disks, tortuotic blood vessels, central scotoma in the visual field, reduced PERG N95, delayed and decreased VEP 100. **(d)** Case 2 at the last check-up 12 years after the disease onset: Pale and atrophic optic disks, decreased visual field scotoma, and thinning of the peripapillary RNFL despite significant VA improvement.

**Figure 2:**
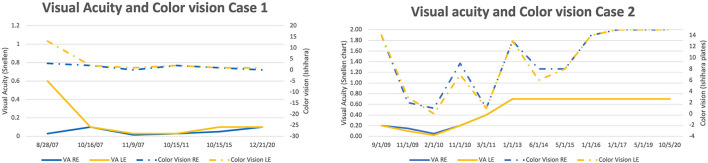
Improvement in VA and color vision, modest in Case 1 and significant in Case 2 with complete recovery of color vision.

A 17-year-old boy was admitted to the eye hospital in September 2007 due to sequential visual acuity (VA) loss, first on the right eye (RE) and 3 weeks later on the left eye (LE). Interestingly, the VA loss on the RE was accompanied by painful eye movements (timeline of the disease is presented in Table 1). In the spring of the same year, he reported an episode of vomiting and dizziness, with vertigo and weight loss. At that time, head computerized tomography (CT) was normal, so he was prescribed antidepressant drugs. Upon admission, VA on the right eye was counting fingers at 3 m, and 0.6 (Snellen) on the LE. Color vision on the RE was poor (1/15) although it was still relatively preserved on his LE 13/15 (Ishihara). Fundoscopy revealed hyperemic optic disks (papilla nervi optici/PNO) without vessel tortuosity and intraretinal hemorrhage at RE superior arcade (Figure 1a). The patient had no early leakage, but some staining of the PNO in the late phases of fluorescein angiography (Supplementary Figure 1A). There was central scotoma in his visual field which increased, with visual acuity decrease and delayed and decreased VEP P100 wave. Pattern ERG N95 wave at that time was still in the normal range, although of lower amplitudes in the worse eye (Figure 1a). The patient was treated with intravenous corticosteroid therapy Solumedrol i.v. (1g/day) for 3 days. Over the next 7 days, his LE visual acuity dropped to 0.3 and was followed by continuous visual function degradation for the next 2.5 months, when it reached the nadir of RLE 0.03 with color vision decreasing to 0/15 and 1/15. 7 years after the onset, visual function started improving with the appearance of the small fenestrations in the central scotoma of both eyes and the patient reached stable 0.1 Snellen bilaterally 14 years after the onset (Figures 1b, 2 and Supplementary Figure 2).

**Table 1:** Medical history and disease progression in the Case 1.

**Date**	**Relevant past medical history**	
Spring 2007	Episode of vomiting and dizziness, with vertigo and weight loss. CT normal, antidepressant drugs prescribed	
	**Summary from initial and follow-up visits**	**Interventions and therapy**
September 2007	VA loss on the RE	Topical anti- glaucomatous medicine
October 2007 (three weeks after the onset of the right eye)	Presentation at Eye Hospital due to VA loss on the LE, Visual acuity RE 0.03, LE: 0.6 Color vision RE 1/15, LE 13/15, central scotoma bilaterally. On fundoscopy hyperemic optic disks, no vessel tortuosity,intraretinal hemorrhage at RE superior arcade. FA: no early leakage, some staining of the PNO in late phases. Delayed and decreased VEP P100 wave. Pattern ERG N95 wave at that time still in normal range	Systemic corticosteroid therapy for 3 days Solumedrol i.v. (1g/day), no improvement
October 2007 (4 weeks after the onset of the right eye)	LE visual acuity dropped to 0.3, RE 0.015	Blood taken for genetic testing
December 2007	Visual acuity 0.1 on both eyes. PERG N95 showed abnormal morphology and abnormal N95/P50 ratio on both eyes. VEP was severely reduced on both eyes and delayed on LE	
January 2008 (2.5 months after the onset on the left eye)	VA reached nadir of RLE 0.02 with color vision decrease to 0/15 and 1/15	Genetic testing came back negative for three common mutations and NGS mtDNA
April 2011	VEP was undetectable on RE and severely reduced on LE. The quality of PERG recording was poor (problems with fixation due to low VA), abnormal N95/P50 ratio was seen on the left eye	
January 2014 (seven years after the onset)	Slight visual function improvement RE 0.1, LE 0.03 with appearance of the small fenestrations in central scotoma of both eyes	
January 2021	Patient reached stable 0.1 Snellen bilaterally 14 years after the onset	Blood taken for genetic testing. Clinical exome negative. After the discovery of the DNAJC 30 causative mutations the sample was retested
September 2021 (14 years after the onset)	Genetical confirmation of the arLHON	

### Case 2

An 18-year-old boy was admitted to our hospital in September 2009 due to bilateral painless visual acuity loss which lasted for 3 weeks (timeline of the disease is presented in Table 2). Upon admission, his RE VA was 0.2 and LE 0.15 on the Snellen chart, whilst color vision was still, surprisingly, 15/15 (Ishihara) on both eyes.

**Table 2:** Medical history and disease progression in the Case 2.

	**Summary from initial and follow-up visits**	**Interventions and therapy**
August 2009	Simultaneous VA loss on the RLE	
September 2009 (three weeks after the onset of the right eye)	Presentation at Eye Hospital due to bilateral VA loss, Visual acuity RE 0.2, LE: 0.15 Color vision RE 15/15, LE 15/15, central scotoma bilaterally. On fundoscopy circumpapillary telangiectatic microangiopathy, swelling of the retinal nerve fiber layer (RNFL). FA: no leakage, EF: decreased N95 wave and delayed and decreased VEP P100	Systemic corticosteroid therapy for 3 days Solumedrol i.v. (1g/day), no improvement. MRI of the head and brain, no signs of demyelination, Aqp4 negative, excluded all other possible causes (infectious, paraneoplastic, compressive, etc.) Blood taken for genetic testing
February 2010 (five months after the onset)	LE visual acuity dropped to nadir RLE 0.02, color vision RE 0/15, LE 1/15, enlargement of the visual field scotoma, amplitude of the N95 decreased, VEP P100 delayed and decreased	Genetic testing for three common mutations and NGS mtDNA negative
November 2010 (14 months after the onset on the left eye)	Improvement of the visual function VA RLE 0.2 with color vision decrease to 0/15 and 1/15, multiple fenestrations in visual field scotoma	
January 2013 (4.5 years after the onset)	Visual acuity reached final level of improvement RLE 0.7, Color vision improvement RLE 15/15, multiple fenestrations in central scotoma of both eyes	
January 2017	VA RLE 0.7, Color vision RLE 15/15, Normal N95 amplitude, but abnormal shape, VEP latency improved, but still prolonged with normal amplitude. Even more fenestrations within visual field scotoma	Blood taken for genetic testing. Clinical exome negative. After the discovery of the DNAJC 30 causative mutations the sample was retested and DNAJC 30 mutation 152 A>G (p.Tyr51Cys) was identified
September 2021	Genetical confirmation of the arLHON	

Fundoscopy was pathognomonic for LHON with circumpapillary telangiectatic microangiopathy, swelling of the retinal nerve fiber layer (RNFL) – pseudo edema, and vessel tortuosity without leakage on fluorescein angiography (Figure 1c and Supplementary Figure 1B). The electrophysiology showed decreased N95 wave and delayed and decreased VEP P100 (Figure 1c). The patient was treated with intravenous corticosteroid therapy Solumedrol i.v. (1 g/day) for 3 days without improvement. Over the next 5 months, his visual acuity further deteriorated to counting fingers at 1 m and his visual field scotoma enlarged. At 14 months, after 9 months of legal blindness, numerous fenestrations in central scotoma appeared and the patient VA improved to 0.2. During the next 3 years, the patient experienced continuous VA improvement. Visual acuity improved to 0.7 (Figure 1d), whilst the visual field has been continuously improving during the follow-up period (Supplementary Figure 3). It is also of interest to note excellent late improvement of color vision.

Both of our patients had genetic testing for LHON after the presentation. They were first tested for three common mutations. Then, next-generation sequencing (NGS) of the mtDNA and NGS of the clinical exome (panel for the optic atrophy mutations known at that time) were carried out, followed by whole exome sequencing, which were all negative. For 14 and 12 years, respectively the patients were without a confirmed diagnosis up until recently when they were retested and DNAJC30:c.152G>A pathogenic homozygous variant c.152A>G (p.Tyr51Cys) in DNAJC30:c.152G>A gene was confirmed.

A short phenotype description of both patients was included in the article by Jarc Vidmar et al. (3) due to some changes in mtDNA which later were not identified as pathogenic.

### Comparison of functional and structural features

Both of our patients had visual acuity improvement which was modest in case 1 (started at year 7 and improved from NADIR off chart to 0.1 during the next 7 years), and significant in case 2 (started at month 14 and continued for the next 2 years to reach stable 0.7 bilaterally). The visual acuity improvement in case 2 was accompanied by an almost complete recovery of the color vision, whilst there was no improvement of color vision in Case 1 (Figure 2). The visual recovery in Case 2 corresponded well to the VA change. During the period of VA deterioration, there was an enlargement of the central scotoma in the visual field. At 14 months, first fenestrations in the visual field appeared and VA started improving (Supplementary Figure 3).

The peripapillary RNFL thickness decreased during the chronic phase in both patients, even with improved and stable visual acuity, and corresponded with the progression of the optic disc atrophy (Supplementary Figures 4, 5). In comparison to the controls, there was significant thinning of 360-degree retinal thickness in both patients (Supplementary Table 2). The greatest thinning was present in superior temporal, temporal, and inferior temporal parts both at 3.5 and 12 years (Supplementary Table 2). When we compared the RNFL thickness between the 3.5 and 12y check-up, Case 1 had an average decrease of the peripapillary RNFL thickness from 47.43 to 44.86 μm RE and from 42.71 to 40.14 μm on the LE. The greatest thinning between 3.5 and 12 y check-ups was noticed in the inferior temporal part of the optic disc (8.77% RE and 13.33 % LE). In Case 2, peripapillary RNFL thickness decreased from an average of 49.14 to 44 μm RE and 51.86 to 43.57 μm LE for all sectors. The greatest thinning was present in the inferior temporal quadrant of the right eye (14.29 %) and the superior temporal quadrant of the left eye (27.87%).

Case 2 had significant improvement in visual function which was also confirmed on microperimetry (Supplementary Figure 6). The first microperimetry was performed at month 14, when visual acuity started to improve. As can be seen, at onset, there was abnormal retinal sensitivity (0 dB) at all spots. The second recording was made 3.5 years after the onset, with the visual acuity bilaterally 0.7 when the mean sensitivity (MS) significantly improved (RE 6.3 dB ± 4.6 dB; range, 0–14 dB; LE 6.5dB ± 6.1dB; range 0–18 dB). The biggest MS improvement was noticed in temporal and nasal macular areas. At the last follow-up, MS somewhat decreased (RE 4.3 dB ± 4.7 dB, range 0–18 dB; LE 4.8 dB ± 5.03 dB, range 0–14 dB). The MS decreased on average RE 2 dB ± 3.9 dB and LE 1.75 dB ± 6.3 dB. Spatially, the most functional were the peripheral regions, especially the nasal and temporal macular area, while the lowest function was detected in the superior and inferior parafoveal region and in the area corresponding to the foveal center (Supplementary Figure 6). Fixation was relatively stable and almost central on all three visits (Supplementary Figure 6).

### Segmentation analysis results

Both of our patients had thinning of the overall retina, RNFL, GCL, and IPL in all ETDRS quadrants in comparison to healthy controls (4), whilst the outer retinal layers were well-preserved in comparison (Supplementary Figures 7–9).

### Electrophysiology results

The delayed and decreased VEP P100 wave in the acute phase of the disease were present in both patients (Figures 1a,c). Case 1 had normal PERG at the first visit, while the N95/P50 ratio became abnormal 3 months later. At the last visit 4 years later, the quality of PERG recording was poor (probably due to problems with fixation caused by low visual acuity), however, an abnormal N95/P50 ratio was seen at least on the left eye, whilst the VEP P100 wave became undetectable. In Case 2, the amplitude of the N95 wave improved, but it remained abnormally shaped and at the level of the isoelectric line; the N95/P50 ratio remained abnormal (under 1), indicating dysfunction of retinal ganglion cells. The amplitude of the P 100 wave improved, but the latency remained delayed (Supplementary Figure 10).

## Discussion

Detailed phenotypic analysis and follow-up of two patients harboring a homozygous DNAJC30:c.152G>A mutation are presented. The affected individuals had profound visual loss at a relatively similar early age (17 and 18 years, respectively). The visual loss was characterized by centrocecal scotoma, abnormal PERG N95 and VEP, and retinal nerve fiber thinning on the OCT. Interestingly, pain in eye movements was present in Case 1, and color vision seemed to be preserved in early phases in both, which is not typical for mtLHON. During the follow-up period, there was an improvement in visual acuity and visual field in both of our patients, although this change was clinically relevant (>0.2 logMAR) (5) only in one case.

The reported time of visual acuity improvement in untreated arLHON patients is 25.8 ± 30.3 months and occurs in ~50% of LHON cases (1). Kieninger et al. reported improvement in 45% of patients with the two novel pathogenic changes in DNAJC30:c.152G>A gene [nonsense variant c.610G>T;p.(Glu204^*^) and the in-frame deletion c.230_232del;p.(His77del)] starting from 1 to 58 months after the onset (median 19 months) (6).

The visual acuity improvement in our patients started at months 84 (Case 1) and 14 (Case 2). This shows that a better prognosis is associated with a faster onset of improvement, but also, that improvement is still possible even after 7 years from the onset.

The thickness of inner retinal layers (RNFL, GCL, and IPL), especially GCL, was decreased in all ETDRS quadrants, with the best preservation in the central circle and in temporal ETDRS quadrants. The significant difference between the two patients was not noticed. INL layer was, on the other hand, thicker in both patients in all quadrants than in healthy controls. This phenomenon was already described first by Carbonelli M. et al. in mtLHON patients with and without macular microcysts (7), and later by Majander et al. in their cohort (8). We report the same observation in our two arLHON patients.

In the acute phase, one patient (Case 2) had a decrease in the PERG N95 wave, reflecting primary ganglion cell dysfunction. This is more specific for LHON than optic neuritis, where ganglion cell deterioration is present only after retrograde degeneration. This is in concordance with our previously published results that suggest that an early decrease in N95 may be a novel biomarker for LHON as it reflects primary cellular energy supply rather than retrograde degeneration (3). A decrease of the PERG N95 amplitude in Case 1 was not present at the first visit (1.5 months after the onset of the disease) but was clearly visible 3 months after. In the chronic phase (3.5 years after the onset of the disease), the amplitude of the VEP P100 wave was undetectable. Parisi et al. (9) reported delayed and decreased VEP P100 which remained unchanged during the follow-up in most of the patients during the 1st year of the disease. In the presented cases, the VEP P100 also remained prolonged and severely decreased during the 1st year. At 14 months with the VA improvement, the VEP P100 latency shortened, and amplitude improved in Case 2. This patient had further improvement of both N95 wave and P100 amplitude at the last check-up, 12 years after the disease onset.

The observed reduction in PERG N95 in the acute phase can be ascribed to an intracellular dysfunction of the innermost retinal layers (RGCs and their fibers). Therefore, the N95 improvement in Case 2 during the follow-up period might correspond to the observed functional improvement.

Barboni et al. (10) showed that, in the acute phase, the pRNFL thickness increases and later on decreases, corresponding to the retinal ganglion cell swelling and apoptosis. In their cohort, pRNFL thinning first occurred in the temporal quadrant, followed by the inferior and superior quadrants, and finally, the nasal quadrant. pRNFL continued to thin slowly in some quadrants even after 60 months, with a significant difference in comparison to the 12–24 months. Stenton et al. (1) also reported peripapillary RNFL thinning in the chronic phase in their cohort of patients harboring the DNAJC30:c.152G>A pathogenic variant. Both of our patients had continuous RNFL thinning during the follow-up, which is in accordance with the results of Wang et al. (11). When we compared Case 1, a patient with modest VA improvement (0.1 Snellen), with Case 2 (0.7 Snellen) we did not find a significant difference in periapillary RNFL thinning, suggesting that functional improvement is not associated with morphological changes. This is somewhat opposite to the findings of Barboni et al. (12), who have found that thinning of the peripapillary RNFL was less prominent in patients with visual acuity recovery. Due to a small number of patients, we cannot conclude whether it is the characteristic of the arLHON or just a random case; further investigation on a larger number of patients is therefore needed.

## Conclusions

Detailed phenotype and long-term follow-up of the first two DNAJC30:c.152G>A patients is presented. Both patients had improvement in VA and visual field over time, one significant and one modest. The color vision, microperimetry, and electrophysiology improvement was present only in the better case. Both patients showed further structural degradation (peripapillary RNFL and segmentation analysis) but stable visual function and relatively good long-term prognosis. Based on the two cases presented, the phenotype of the autosomal recessive LHON is similar to mitochondrial LHON in both early (subacute and dynamic) and (late)chronic phase. It appears that the disease follows the same patterns of the GCC complex and pRNFL thinning described for the mitochondrial LHON, but with a better visual prognosis in some cases.

## Data availability statement

The original contributions presented in the study are included in the article/Supplementary material, further inquiries can be directed to the corresponding author/s.

## Ethics statement

Written informed consent was obtained from the individual(s) for the publication of any potentially identifiable images or data included in this article.

## Author contributions

Patients were clinically characterized by MSH, MJ-V, AF, and SP. Genetic analysis was done by BP, MV, and AM. Electrophysiology was done by JB and MSH. The first draft of the manuscript was written by SP. All authors contributed to the study conception and design, contributed to material preparation, data collection and analysis, commented on previous versions of the manuscript, read, and approved the final manuscript.

## Funding

This study was supported by the research program P3-0333 of the Slovenian Research Agency.

## Conflict of interest

The authors declare that the research was conducted in the absence of any commercial or financial relationships that could be construed as a potential conflict of interest.

## Publisher's note

All claims expressed in this article are solely those of the authors and do not necessarily represent those of their affiliated organizations, or those of the publisher, the editors and the reviewers. Any product that may be evaluated in this article, or claim that may be made by its manufacturer, is not guaranteed or endorsed by the publisher.
