# Supplementary figures and images for: Case report: Long-term follow-up of two patients with LHON caused by DNAJC30:c.152G>A pathogenic variant-case series
Source: Front Neurol. 2022 Oct 28;13:1003046. doi: 10.3389/fneur.2022.1003046 (PMC9649972; doi:10.3389/fneur.2022.1003046)

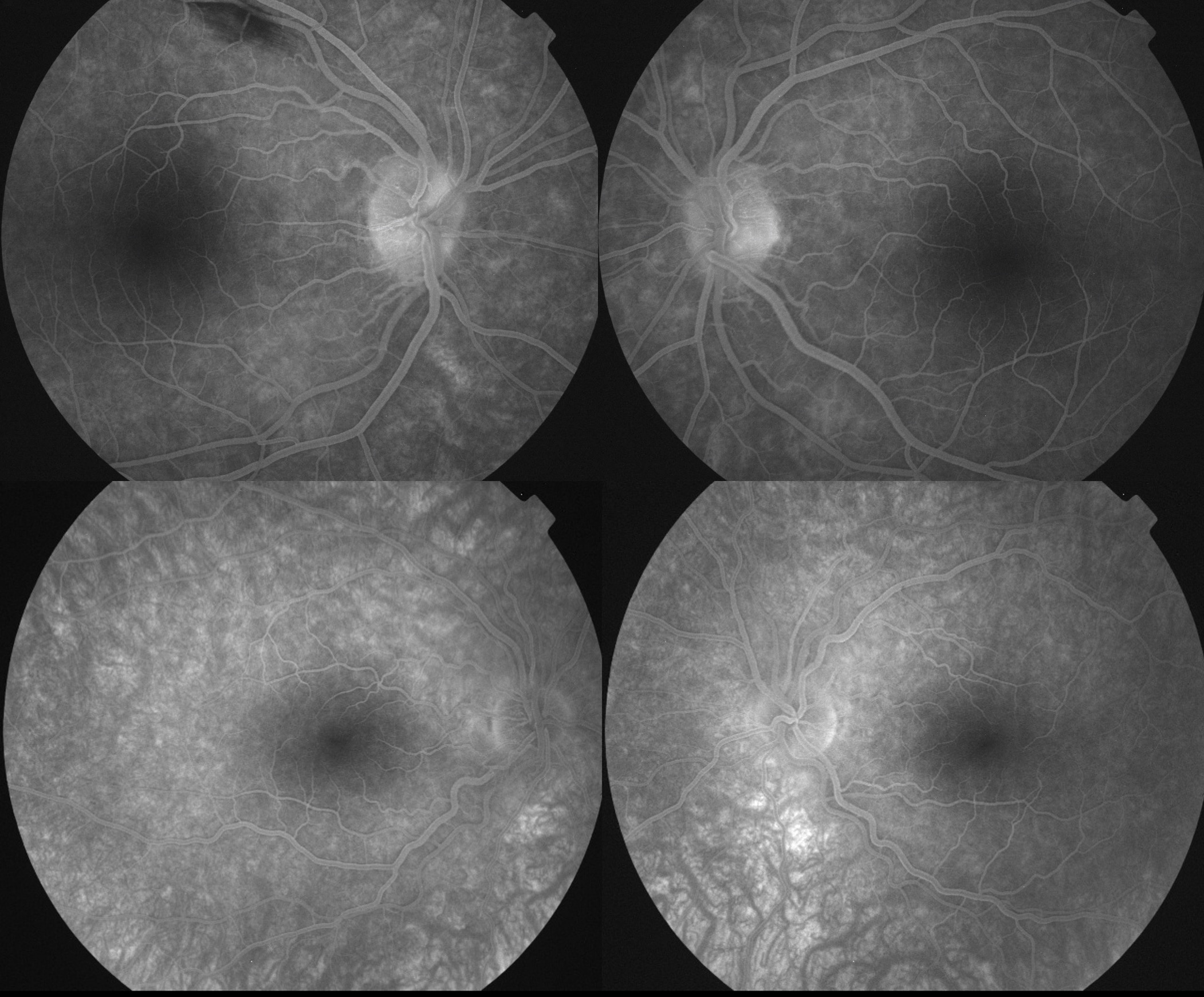

Supplement: Supplementary file 1 [file Image_1.JPEG]

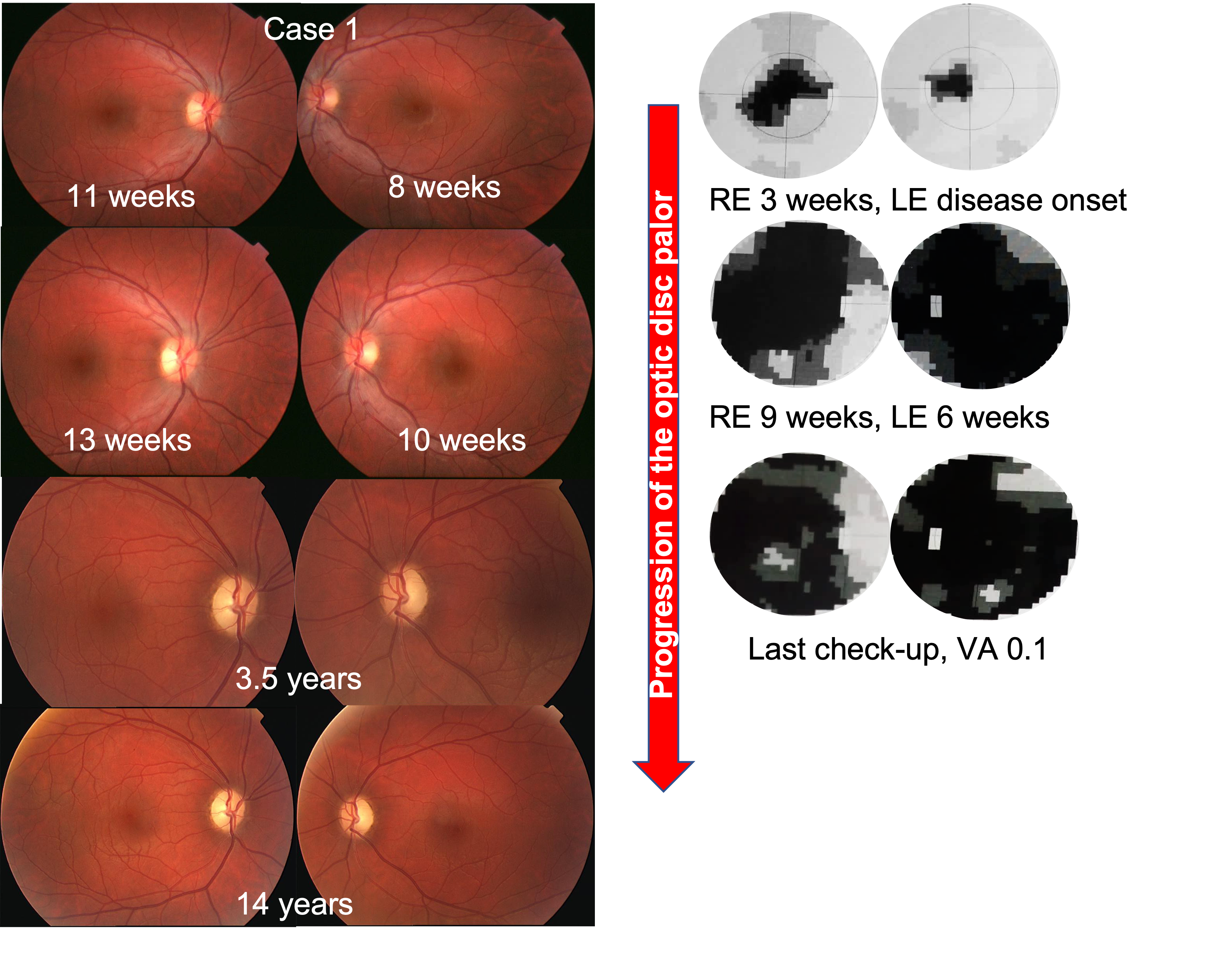

Supplement: Supplementary file 2 [file Image_2.PNG]

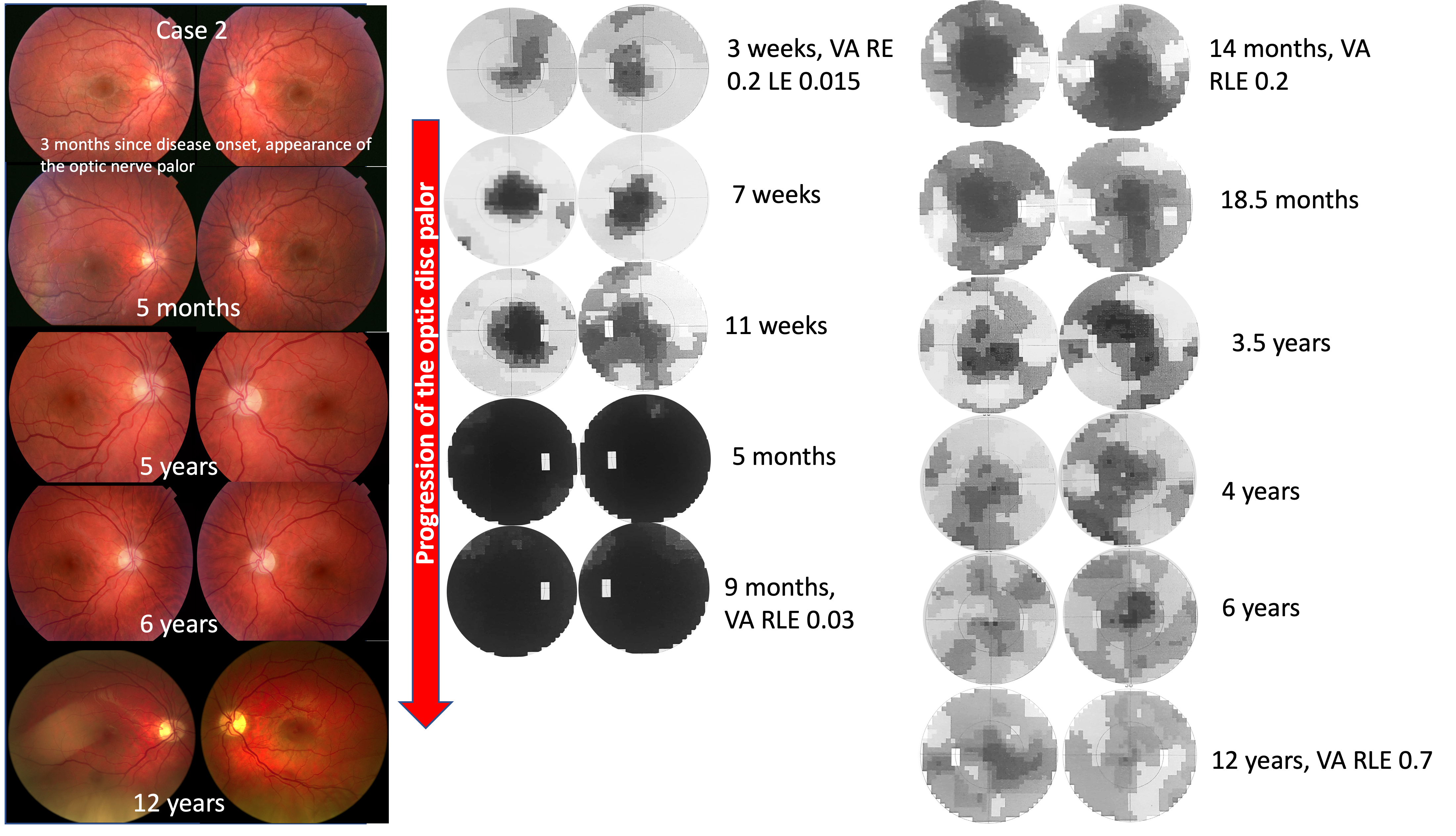

Supplement: Supplementary file 3 [file Image_3.PNG]

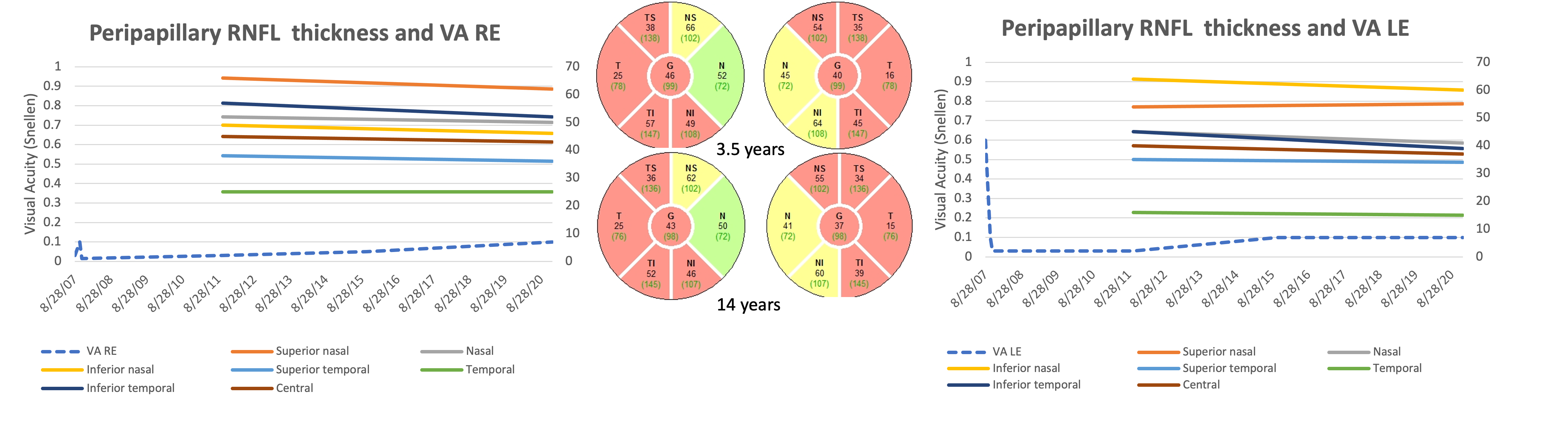

Supplement: Supplementary file 4 [file Image_4.PNG]

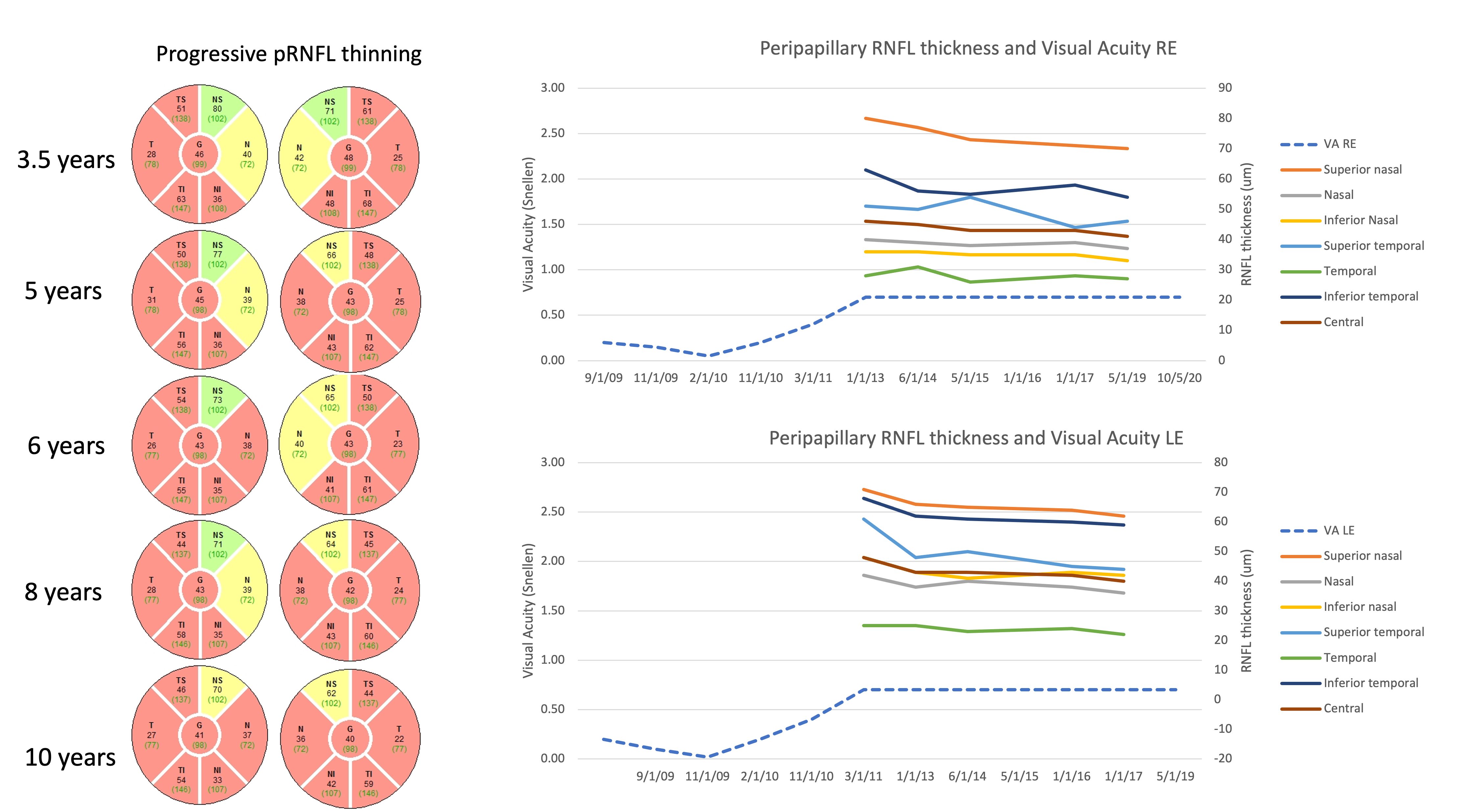

Supplement: Supplementary file 5 [file Image_5.PNG]

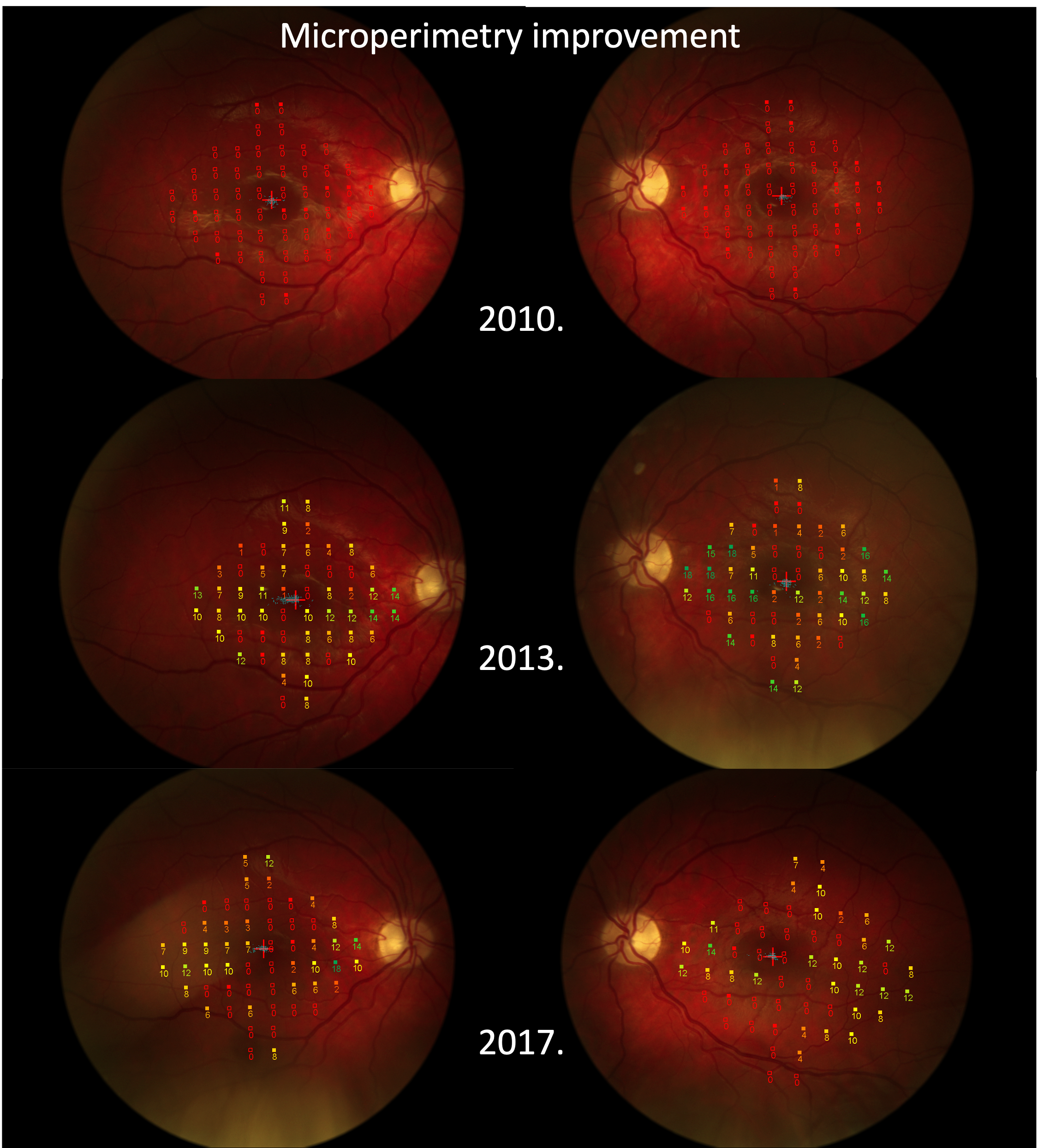

Supplement: Supplementary file 6 [file Image_6.PNG]

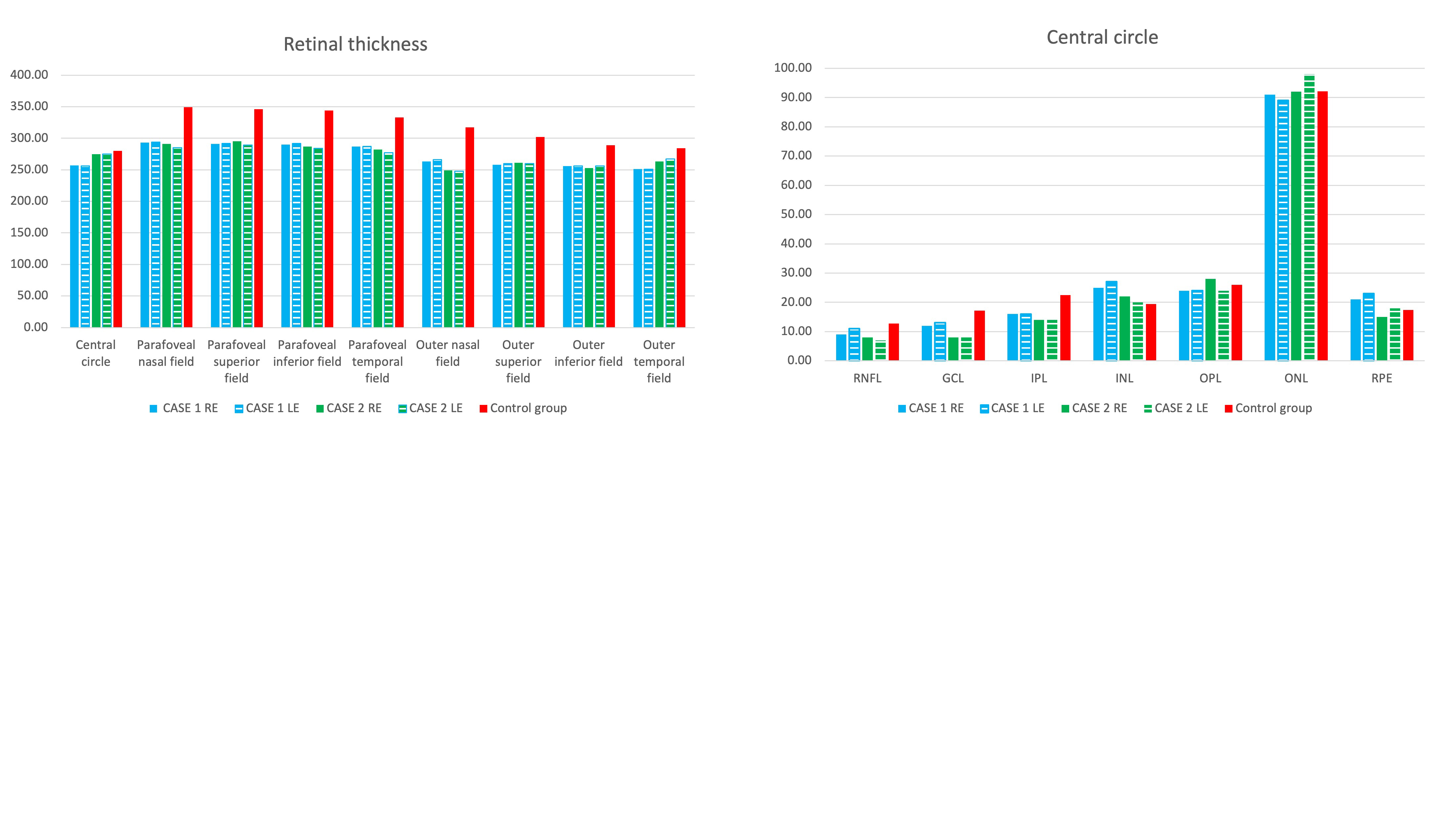

Supplement: Supplementary file 7 [file Image_7.JPEG]

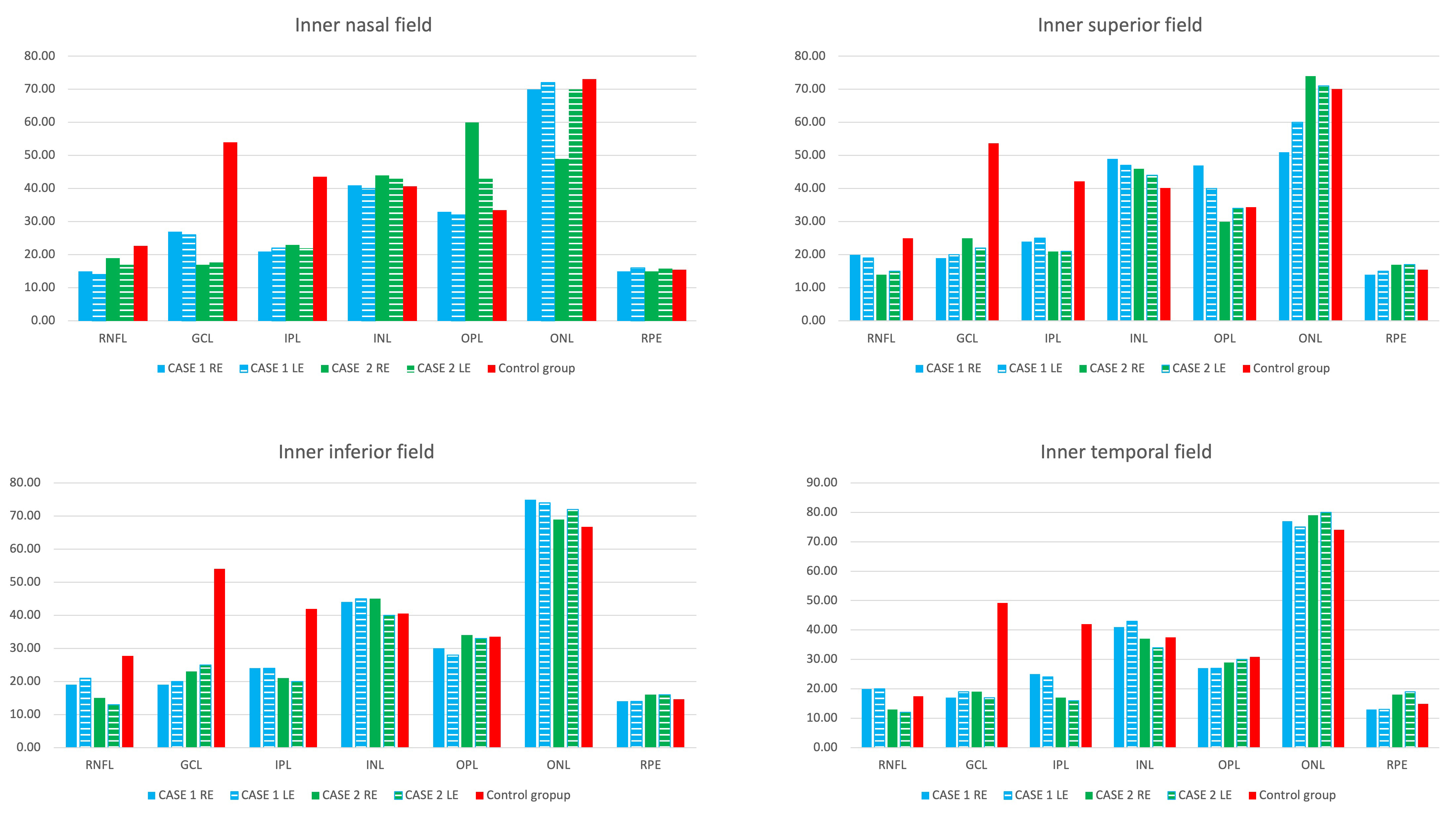

Supplement: Supplementary file 8 [file Image_8.JPEG]

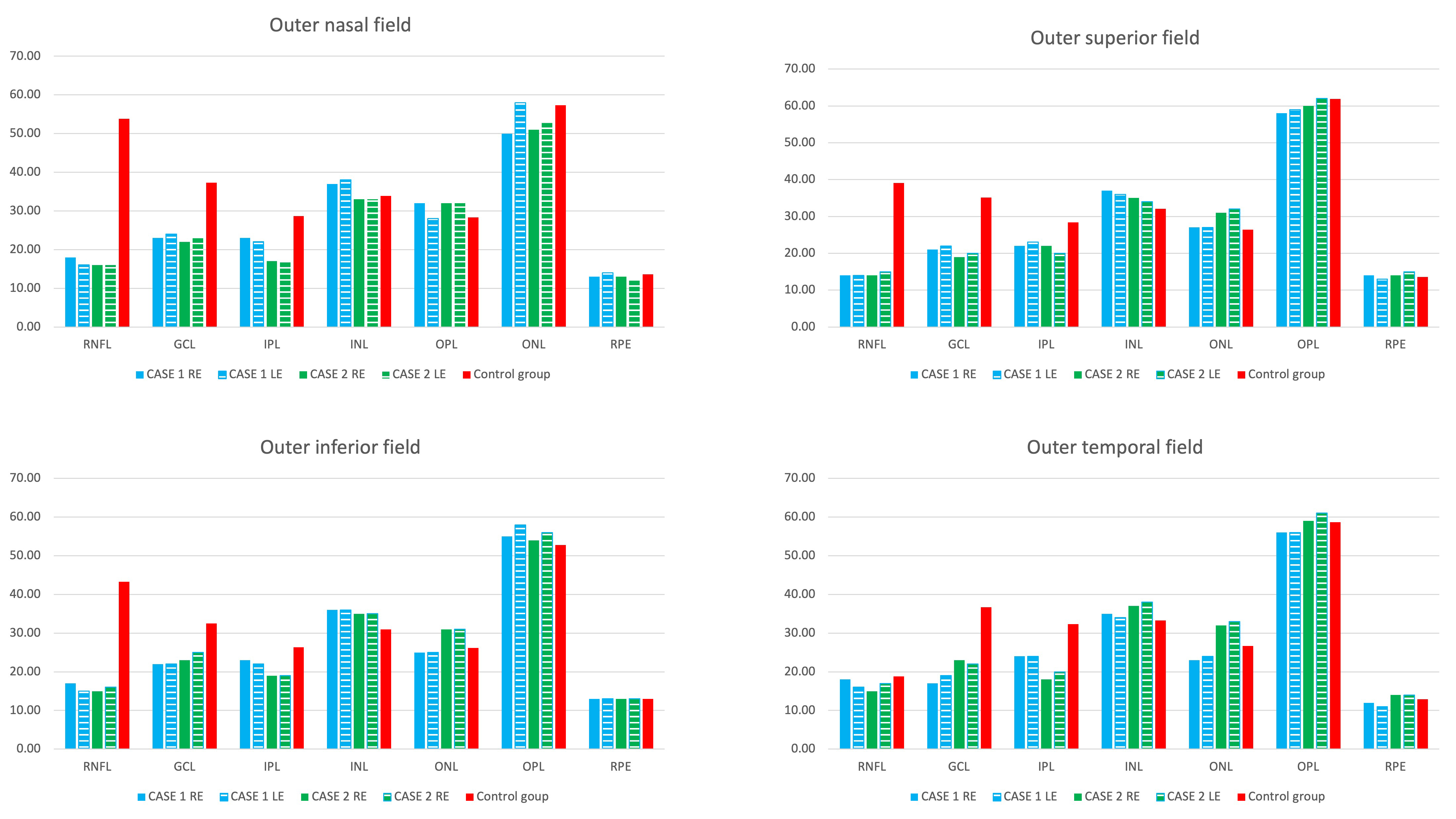

Supplement: Supplementary file 9 [file Image_9.JPEG]

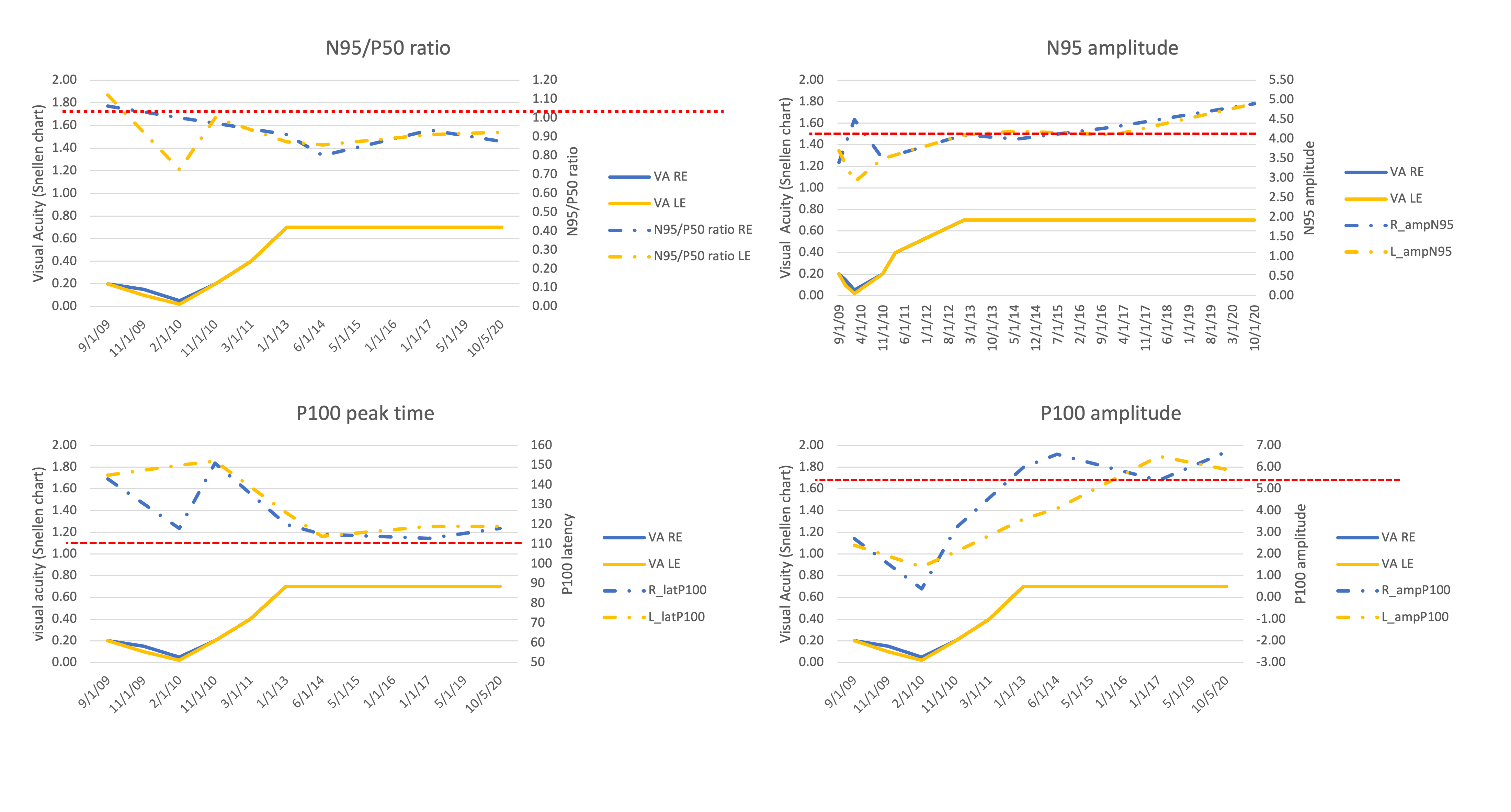

Supplement: Supplementary file 10 [file Image_10.PNG]
