# Supplementary material for: Case report: Long-term follow-up of two patients with LHON caused by DNAJC30:c.152G>A pathogenic variant-case series
Source: Front Neurol. 2022 Oct 28;13:1003046. doi: 10.3389/fneur.2022.1003046 (PMC9649972; doi:10.3389/fneur.2022.1003046)
Supplement: Supplementary file 11 [file Table_1.DOCX]

Methodology

Ring analysis of the retinal nerve fiber layer around optic disc with spectral domain optical coherence tomography (SD-OCT) and segmentation of the retinal layers was done at follow up visits. SD-OCT segmentation was performed by the Spectralis HRA apparatus (Heidelberg Engineering, Heidelberg, Germany) according to the protocol described previously (3). The software automatically provided an Early Treatment Diabetic Retinopathy Study (ETDRS) retinal thickness map by layers in accordance with the consensus of the International Nomenclature for OCT (4). Inadequately defined layers were manually repaired. The results were compared with the published standards for the same machine (3).

The pRNFL thickness was measured using Spectralis HRA+OCT (Heidelberg Engineering, Heidelberg, Germany). Measurements were repeated when the imaging quality was poor. The Spectralis HRA+OCT software automatically calculated all OCT data.

Electrophysiological tests: The large-field (21.6x27.8° screen, 0.8° checkerboard) pattern electroretinogram (PERG) and the visual evoked potentials (VEP) were recorded from both eyes with Espion Diagnosis system (Diagnosys LLC, Littleton, MA, USA) according to the previously described protocol, electrophysiology results of our patients have been described previously (Table 1, LHON 1 and LHON 4) (5).

Static microperimetry was performed after pupil dilation with topical 1% tropicamide in one patient only according to the protocol described previously (4). The grid (Humphrey 10–2) tested 56 retinal locations in the central 20°, with 2° resolution at threshold sensitivities from 0 to 20 dB. Stimuli of the size of Goldmann III target appeared for 200ms and changed intensity in 4–2 strategy. The mean sensitivity (MS) of the whole tested area was calculated using the MP1 software. The preferred retinal locus for fixation (PRL) and fixation stability was recorded during the exam. The fixation stability was determined as the percentage of fixation points that centered inside the 2° or 4° area. These areas were determined by the MP1 software and were centered at the highest density of fixation points.

1. Invernizzi, A, Pellegrini M, Acquistapace A, Benatti E, Erba S, Cozzi M, et al. Normative Data for Retinal-Layer Thickness Maps Generated by Spectral-Domain OCT in a White Population. Ophthalmology Retina (2018) 2:808-15.

2. Staurenghi G, Sadda S, Chakravarthy U, Spaide RF. International Nomenclature for Optical Coherence Tomography (IN•OCT) Panel. Proposed lexicon for anatomic landmarks in normal posterior segment spectral-domain optical coherence tomography: the IN•OCT consensus. Ophthalmology. (2014) 12:1572-78.

3. Jarc-Vidmar M, Tajnik M, Brecelj J, Fakin A, Sustar M, Naji M, et al. Clinical and electrophysiology findings in Slovene patients with Leber hereditary optic neuropathy. Doc Ophthalmol. (2015) 130:179-87.

4. Lumi X, Petrovic Pajic S, Sustar M, Fakin A, Hawlina M. Autologous neurosensory free-flap retinal transplantation for refractory chronic macular hole-outcomes evaluated by OCT, microperimetry, and multifocal electroretinography. Graefes Arch Clin Exp Ophthalmol. (2021) 259:1443-1453.
