# Supplementary material for: Case report: Long-term follow-up of two patients with LHON caused by DNAJC30:c.152G>A pathogenic variant-case series
Source: Front Neurol. 2022 Oct 28;13:1003046. doi: 10.3389/fneur.2022.1003046 (PMC9649972; doi:10.3389/fneur.2022.1003046)
Supplement: Supplementary file 12 [file Table_2.DOCX]

|  |  | PERIPAPILLAR RNFL THICKNESS 3.5Y | | PERIPAPILLAR RNFL THICKNESS 12Y | | PERIPAPILLAR RNFL THICKNESS CONTROLS | THICKNESS REDUCTION (%) 3.5Y | | THICKNESS REDUCTION (%) 12Y | |
| --- | --- | --- | --- | --- | --- | --- | --- | --- | --- | --- |
|  | QUADRANT | RE | LE | RE | LE |  | RE | LE | RE | LE |
| Case 1 | SUPERIOR NASAL | 66.00 | 54.00 | 62.00 | 55.00 | 102 | 35.29 | 47.06 | 39.22 | 46.08 |
|  | NASAL | 52.00 | 45.00 | 50.00 | 41.00 | 72 | 27.78 | 37.50 | 30.56 | 43.06 |
|  | INFERIOR NASAL | 49.00 | 64.00 | 46.00 | 60.00 | 107 | 54.21 | 40.19 | 57.01 | 43.92 |
|  | SUPERIOR TEMPORAL | 38.00 | 35.00 | 36.00 | 34.00 | 137 | 72.27 | 74.45 | 73.72 | 75.18 |
|  | TEMPORAL | 25.00 | 16.00 | 25.00 | 15.00 | 77 | 67.53 | 79.22 | 67.53 | 80.52 |
|  | INFERIOR TEMPORAL | 57.00 | 45.00 | 52.00 | 39.00 | 146 | 60.96 | 69.18 | 64.38 | 73.29 |
|  | AVERAGE | 45.00 | 40.00 | 43.00 | 37.00 | 98 | 54.08 | 59.18 | 56.12 | 62.24 |
| Case 2 | SUPERIOR NASAL | 80.00 | 71.00 | 70.00 | 62.00 | 102 | 21.57 | 30.39 | 31.37 | 39.22 |
|  | NASAL | 40.00 | 42.00 | 37.00 | 36.00 | 72 | 44.44 | 41.67 | 48.61 | 50.00 |
|  | INFERIOR NASAL | 36.00 | 48.00 | 33.00 | 42.00 | 107 | 66.36 | 55.14 | 69.16 | 60.75 |
|  | SUPERIOR TEMPORAL | 51.00 | 61.00 | 46.00 | 44.00 | 137 | 62.77 | 55.47 | 66.42 | 67.88 |
|  | TEMPORAL | 28.00 | 25.00 | 27.00 | 22.00 | 77 | 63.64 | 67.53 | 64.94 | 71.43 |
|  | INFERIOR TEMPORAL | 63.00 | 68.00 | 54.00 | 59.00 | 146 | 56.85 | 53.42 | 63.01 | 59.59 |
|  | AVERAGE | 46.00 | 48.00 | 41.00 | 40.00 | 98 | 53.06 | 51.02 | 58.16 | 59.18 |

Supplementary Table 1 Peripapillary RNFL changes at 3.5- and 12-years check-up
