# Supplementary material for: Case report: Long-term follow-up of two patients with LHON caused by DNAJC30:c.152G>A pathogenic variant-case series
Source: Front Neurol. 2022 Oct 28;13:1003046. doi: 10.3389/fneur.2022.1003046 (PMC9649972; doi:10.3389/fneur.2022.1003046)
Supplement: Supplementary file 13 [file Table_3.DOCX]

Supplementary Figure legend

Supplementary Figure 1: (**A**) Fluorescein angiography of both eyes of Case 1 showing staining of the optic disc on both eyes (**B**) Fluorescein angiography of both eyes of Case 2 shows no leakage on the optic disc

Supplementary Figure 2: Progression in optic disc atrophy during the follow-up period and appearance of the small fenestrations in the central scotoma in Case 1

Supplementary Figure 3: Progression in optic disc atrophy during the follow-up period and improvement of the visual field in Case 2

Supplementary Figure 4: Slight thinning of the peripapillary RNFL during the follow-up period despite small visual acuity improvement (dashed blue line) during the follow-up period in Case 1

Supplementary Figure 5: Progressive thinning of the peripapillary RNFL in all quadrants during the follow-up period despite significant visual acuity improvement (dashed blue line) during the follow-up period in Case 1

Supplementary Figure 6: Microperimetry of Case 2 showing improvement in sensitivity, especially in the temporal and nasal part of the macula

Supplementary Figure 7: (**A**) Retinal thickness in different ETDRS quadrants in Case 1, Case 2, and Control group (**B**) Thickness of the different retinal layers in the central ETDRS circle in Case 1, Case 2, and Control group

Supplementary Figure 8: Thickness of the different retinal layers in temporal, nasal, superior and inferior inner ETDRS fields in Case 1, Case 2, and Control group

Supplementary Figure 9: Thickness of the different retinal layers in temporal, nasal, superior and inferior outer ETDRS fields in Case 1, Case 2, and Control group

Supplementary Figure 10: Electrophysiology results in Case 2 show normalization of the N95 amplitude value, but N95/P50 ratio remains abnormal, under 1, which is in concordance with the noticed abnormal shape of the N95. VEP P 100 wave amplitude value normalized as well, but peak time remained prolonged despite visual acuity improvement.
